# Proton Dipolar Spin–Lattice Relaxation in Nano-channels of Natrolite

**DOI:** 10.1007/s00723-016-0805-5

**Published:** 2016-06-25

**Authors:** M. Paczwa, A. A. Sapiga, M. Olszewski, N. A. Sergeev, A. V. Sapiga

**Affiliations:** Department of Mathematical and Physics, Institute of Physics, University of Szczecin, Wielkopolska str.-15, 70-451 Szczecin, Poland; V.I.Vernadsky Crimean Federal University, 295007 Simferopol, Crimea

## Abstract

The ^1^H nuclear magnetic resonance (NMR) spectra and the dipolar spin–lattice relaxation time *T*_1D_ for ^1^H in the natural natrolite (Na_2_Al_2_Si_3_O_10_·2H_2_O) have been measured in the temperature range of 190–390 K. From the temperature transformations of ^1^H NMR spectra, it follows that at *T* > 300 K, the diffusion of water molecules along the nano-channels is observed. From experimental *T*_1D_ data, it follows that the 180° flip motion of the water molecules takes place in natrolite. At low temperature (*T* < 250 K), the dipolar interaction with paramagnetic impurities as a relaxation mechanism of ^1^H nuclei becomes significant.

## Introduction

**Fig. 1:**
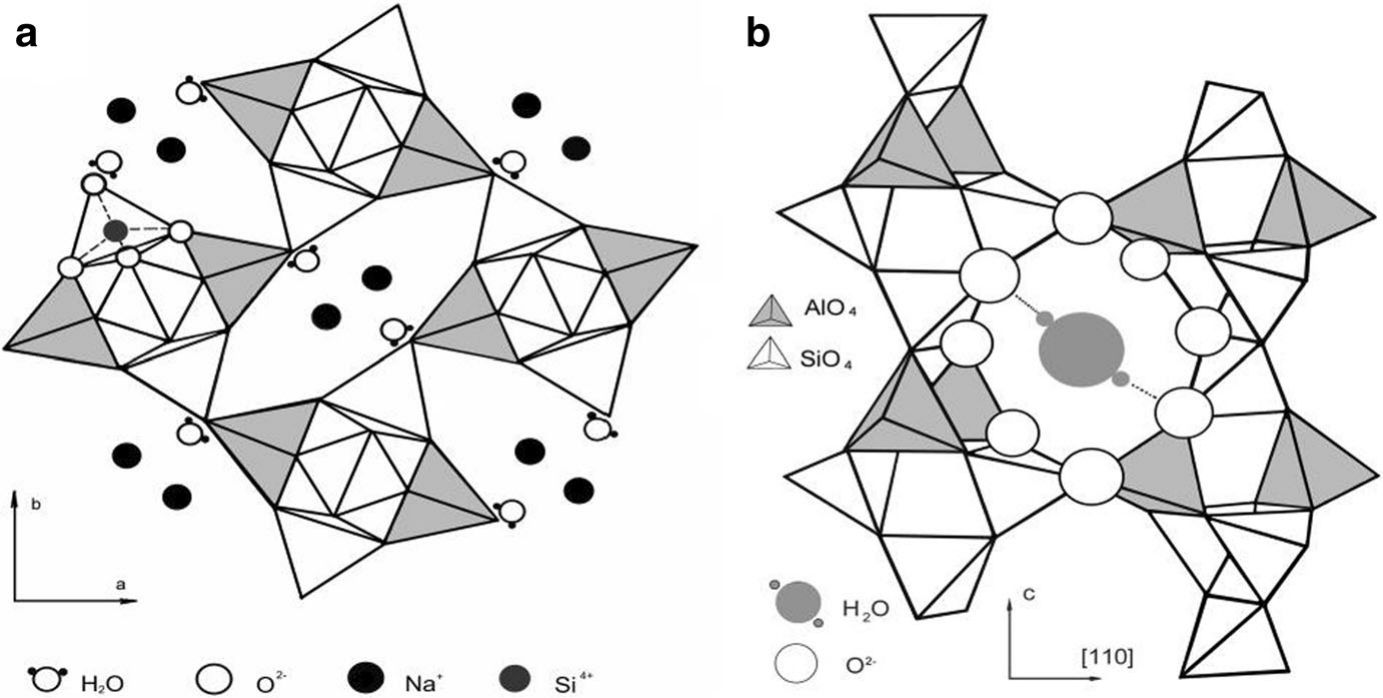
Natrolite structure. **a** Projected on the (001) plane. **b** The natrolite chains and window from oxygen ions

Physical properties of materials entrapped in nano-sized cavities of significant interest for both fundamental science and application of nano-porous compounds. The mineral natrolite is a typical porous compound (zeolite) with the narrow nano-channels [1]. The natrolite structure careful refinements carried out by X-ray and neutron diffraction [2–5]. The natrolite unit cell is orthorhombic with space group Fdd2 and contains eight formula units Na_2_Al_2_Si_3_O_10_·2H_2_O. The natrolite framework consists of tetrahedra of alumina (AlO_4_) and silica (SiO_4_) chains linked together via common oxygen atoms. The natrolite structure contains channels running parallel to the c-axis (Fig. 1a) and channels connected among themselves by oxygen windows (Fig. 1b). These oxygen rings create a system of channels, which are placed perpendicular to c-axes and are crossed a framework approximately along of directions of a type [110].

**Fig. 2:**
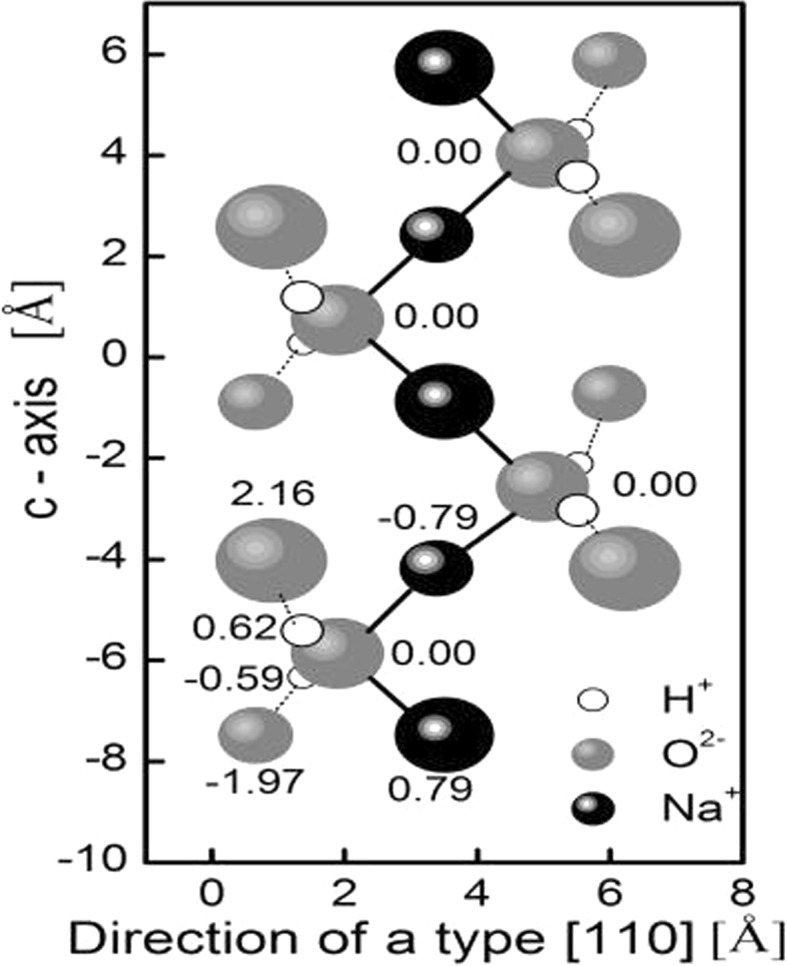
Natrolite structure projection on a water molecules plane. The *solid lines* are electrostatic bonds between oxygen of water and sodium ions. The *dotted lines* are the hydrogen bonds. The *numbers* specify height of ions from the plane

The water molecules and sodium ions form zigzag chains along the channel parallel to c-axis. Water molecules occupy two sites in the channels and the other sites are occupied by sodium ions. Each water molecule is coordinated by two framework oxygen atoms and by two sodium ions (Fig. 2).

One of the main features of natrolite is a narrow diameter of channels. The diameter of the oxygen’s windows in the [110] direction is 2.60 A, but the diameter of channel paralleled to the c-axis is only 2.08 A [2], and it is less than 2.8 A, which is usually assumed for the diameter of water molecule [6]. Therefore, the interesting problem is the determination of the microscopic mechanism of water molecules mobility in the natrolite channels.

The mobility of the water molecules in natrolite was studied by the ^1^H nuclear magnetic resonance (NMR) method in [7–15]. The first research of molecular mobility in natrolite by a method was realized by Gabuda [7, 8]. In this paper, it was assumed that the main kind of mobility of water molecules at high temperature in natrolite is the diffusion along channels parallel to c-axis and water molecule jumps without breaking of one chemical bond of water molecule with sodium ion. Later from NMR data, it was found that at high temperature of 363 K, there is diffusion of water molecules along c-channels, and at temperature higher than 443 K, there is also diffusion in the perpendicular channels [9–13]. From the analysis of the temperature transformations of ^1^H NMR spectra, it has been obtained that the water molecules diffuse only along the regular positions (the Schottky defects), which coincide with positions of water molecules in a rigid lattice [10]. This motion is the “hopping” motion, i.e., the water molecules spend most of their time in a potential well corresponding to equilibrium positions, and only a very small fraction moves between these potential wells. Thompson et al. [9] from analyses of the temperature transformations of ^1^H spin–lattice relaxation times in the laboratory *T*_1_ and rotating *T*_1ρ_ frames assumed that the 180° flip motion of the water molecules takes place in natrolite. From our experimental *T*_1_ and *T*_1ρ_ data, it follows that at *T* > 250 K, the diffusion of water molecules in parallel and perpendicular to the c-axis channels of natrolite is observed [15]. Our ^23^Na and ^27^Al NMR investigations show that in temperature range up to 573 K, the phase transitions in natrolite, connected with modifications of the aluminosilicate framework or the structure of pores, are not observed [13, 16, 17]. It follows also from these data that the diffusions of sodium cations in natrolite are absent.

In solid insulators containing spin-1/2 nuclei, spin–lattice relaxation in a laboratory frame is usually dominated by the dipole–dipole interaction among nuclear spins [18]. Temperature dependence of the relaxation times provides important information on molecular dynamics. Moreover, the secular part of the dipole–dipole interactions that commutes with the Zeeman term forms an independent energy reservoir [19–21] with its own spin temperature, which is different from the spin temperature of the Zeeman reservoir. Next, dipole–dipole interaction plays an important role in spin diffusion, which brings a nuclear-spin system into a thermal equilibrium with the lattice by means of nuclear spin interaction with paramagnetic impurities [20, 21].

It should be noted that it is well known that the proton spin–lattice relaxation time, *T*_1_, in laboratory frame is sensitive to molecular motions with frequencies of the order of Larmor frequency *ω*_0_ or in the MHz region [18]. However, the spin–lattice relaxation time, *T*_1D,_ of the dipolar reservoir is sensitive to slow molecular motion in the kHz region.

In this paper, we have investigated the mobility of the water molecules in nano-channels of natrolite by the ^1^H NMR pulse method, measuring the temperature dependences of ^1^H spectra and the spin–lattice dipolar relaxation time *T*_1D_.

## Experiment Procedure

A polycrystalline sample of natural natrolite from Khibiny deposit (Kola Peninsula, Russia) was used for the NMR measurements [13]. The ^1^H NMR spectra and spin–lattice relaxation times were measured at ν_0_ = 400.13 MHz frequency in 9.4 T magnetic field using a Bruker Avance-400 NMR spectrometer. The dipolar relaxation time, *T*_1D,_ was measured using a modified Jeener–Broekaert sequence with phase cycling: *π*/2(*x*) − *τ*_1_ − *π*/4(*y*) − *τ* − *π*/4(*y*) − *τ*_2_ − acq( −*y*) and *π*/2( −*x*) − *τ*_1_ − *π*/4(*y*) − *τ* − *π*/4(*y*) − *τ*_2_ − acq(*y*) [22]. Here, acq(±*y*) are the signal acquisitions in the ±*y* of *y* directions; the phase of the pulses is shown in the parentheses. The time *τ*_1_ between the initial *π*/2 pulse and the following *π*/4 pulse was adjusted to produce the maximal Jeener signal, and the time *τ* between the *π*/4 pulses was varied to determine the decay of the dipolar order.

## Results and Discussion

**Fig. 3:**
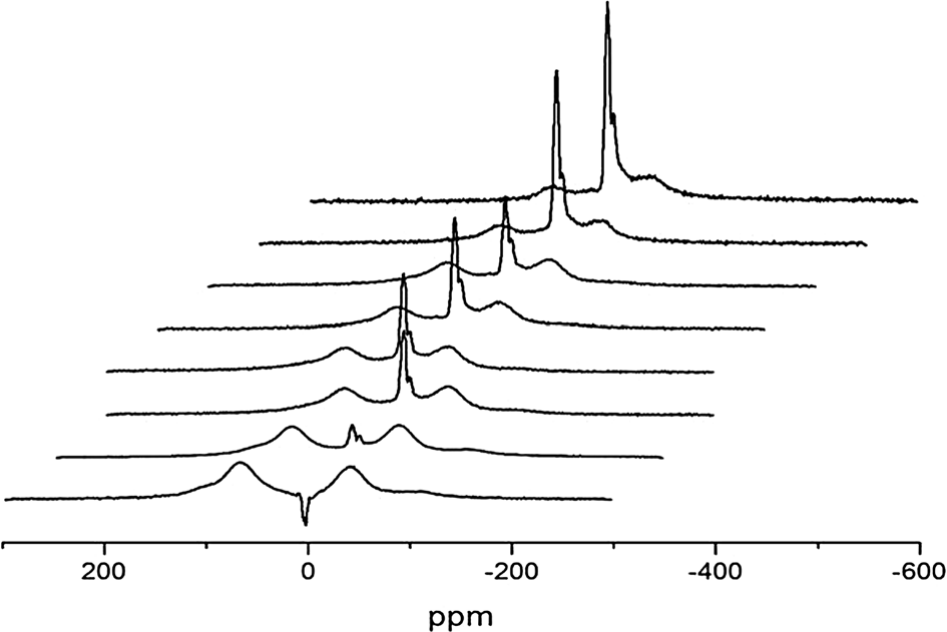
^1^H NMR spectra of polycrystalline natrolite at different temperatures obtained by the solid-echo method [23]. The temperatures in K of the sample, counting from *bottom* to *top*, are as follows: 200; 310; 315; 320; 325; 330; 340; and 350

^1^H NMR spectra of natrolite obtained by the solid-echo method [23] at different temperature are shown in Fig. 3.

**Fig. 4:**
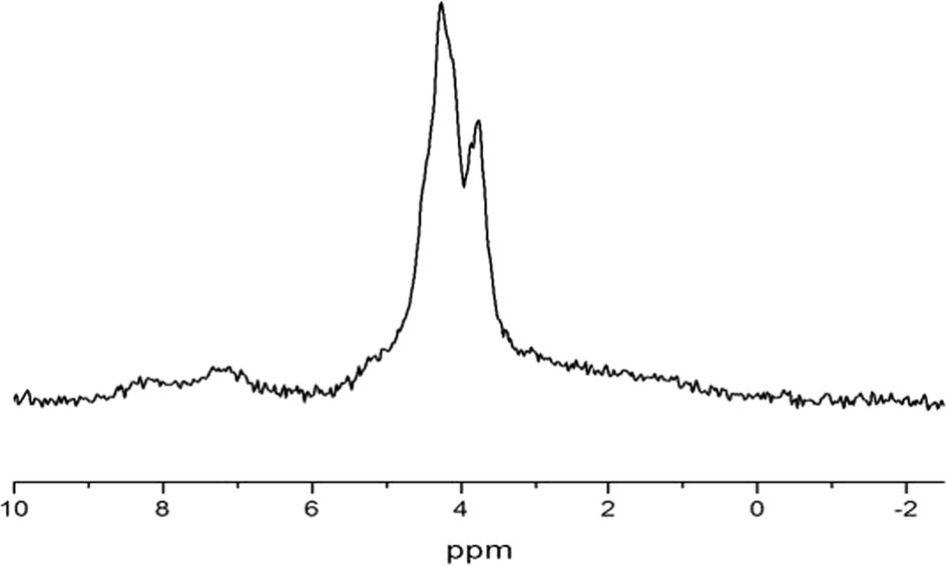
^1^H MAS NMR spectrum of natrolite at temperature *T* = 300 K and at rotational frequency *Ω*_rot_ = 5 kHz

At temperatures *T* < 300 K, the ^1^H spectra are represented by broad Pake doublets [24], meaning that translational and rotational mobility of water molecules in the natrolite channels is completely frozen and that the water molecules form a “rigid” quasi-one-dimensional lattice in the wide channels. In additional to broad Pake doublets, a central single line is observed, which is presumably coming from some amount of hydroxyl (OH^*·*−^) ions, the characteristic of zeolites. The experimental ^1^H MAS NMR spectrum of natrolite is shown in Fig. 4.

The rotation of the sample at magic angle (MAS NMR) leads to the full averaging of dipolar interactions between magnetic moments of ^1^H nuclei, and the shape of NMR spectra is determined by the isotropic electron–nuclear interactions (chemical shifts). Therefore, from Fig. 4, we see that in natrolite, there are two different ^1^H nuclei with different chemical shifts. The line in MAS NMR spectrum at frequency <4 ppm is related to hydroxyl *OH*^−^ ions and the line at frequency >4 ppm is connected with protons of the water molecules.

**Fig. 5:**
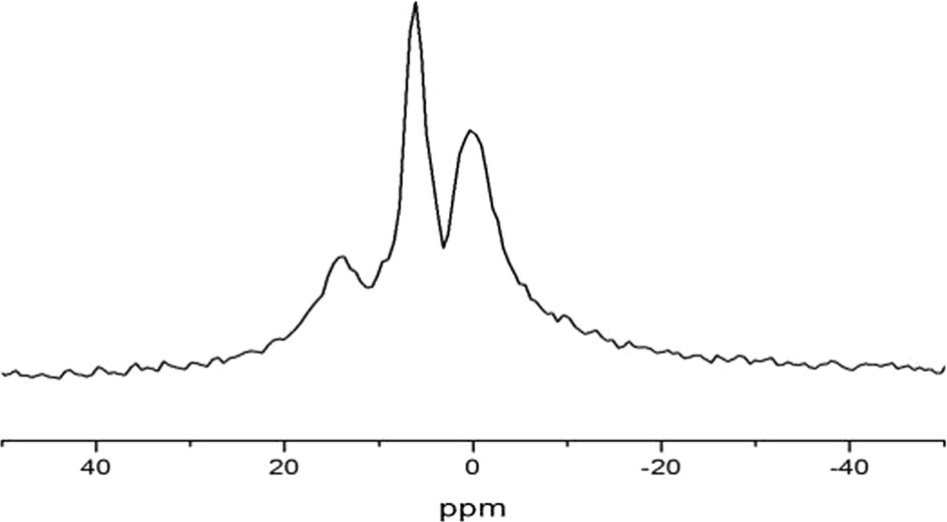
^1^H NMR spectra of polycrystalline natrolite at *T* = 380 K obtained by the solid-echo method [23]

At temperatures above 350 K, the ^1^H NMR spectra (without rotation of sample) are transformed into a narrow doublet and a central single line (Fig. 5).

Such transformation is often observed for fast anisotropic (in the case in question, quasi-one-dimensional) molecular diffusion in zeolite nano-channels, which results in effective motional averaging of the intermolecular dipole–dipole interactions of nuclear spins, while the averaging of the intramolecular dipole–dipole interactions is only partial. This results in a distinct fine structure of the NMR spectrum [25, 26].

It should be noted that at temperature 380 K, the fine structure of the spectra is not lost, and a single central Lorentzian-like line is also observed. This fact can be explained, suggesting that the proton exchange between water molecules and hydroxyl groups is not observed in natrolite. The proton exchange between the water molecules and hydroxyl ion must leads to the lost of the fine structure of the NMR spectra and a central single Lorenzian-like line must vanish. However, from Fig. 5, we see that a central single Lorenzian-like line in ^1^H NMR spectrum has remained.

**Fig. 6:**
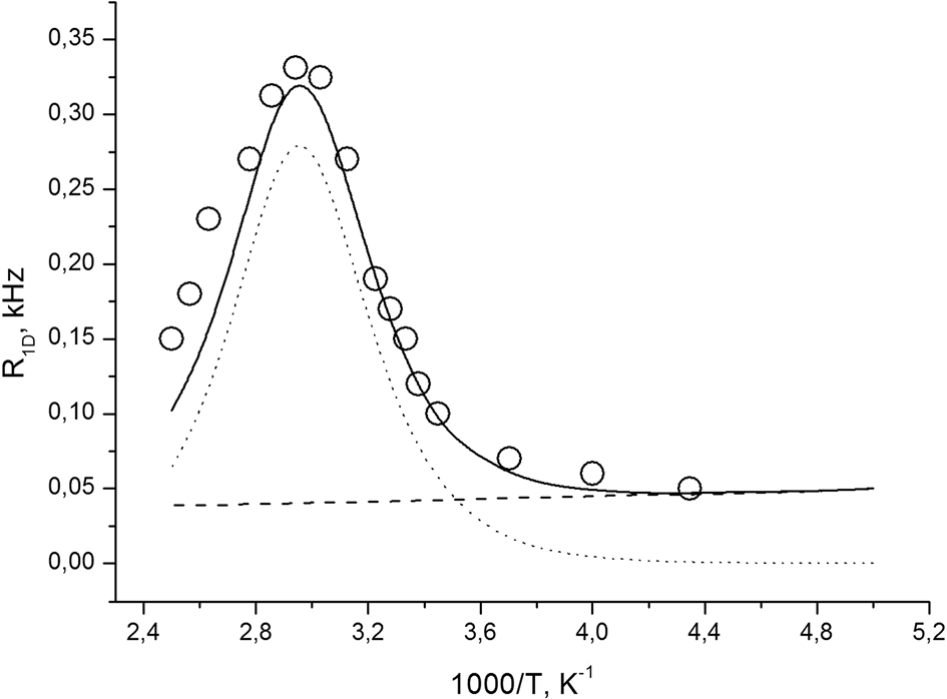
Temperature dependence of the dipolar spin–lattice relaxation rate, *R*_1D_ = 1/*T*_1D_, in polycrystalline natrolite. The experimental data are illustrated by *circles*; the theoretical data are indicated by a *straight line*. The theoretical dependence of *R*_1D‹*d*›_ = 1/*T*_1D‹*d*›_ is indicated by a *dotted line*. The theoretical dependence of *R*_1D‹*ne*›_ = 1/*T*_1D‹*ne*›_ is indicated by a *dashed line*

Next, let us discuss our Jeener–Broekaert experiment [19] that transforms the spin system from Zeeman order characterized by spin alignment along the external magnetic field into the dipolar order with alignment of spins in the local magnetic field produced by their neighbors. In this case, the system is characterized by the dipolar relaxation time, *T*_1D_, which describes the spin relaxation of the dipolar reservoir. Temperature dependence of the ^1^H dipolar relaxation rate $R_{{ 1 {\text{D}}}} = T_{{ 1 {\text{D}}}}^{ - 1}$ is given in Fig. 6.

It should be noted above that the proton spin–lattice relaxation of the dipolar reservoir is sensitive to molecular motion with frequencies of the order of a frequency $\omega_{\text{L}} = \gamma B_{\text{L}}$, where

1 $$B_{\text{L}} = \sqrt {\frac{{5M_{{ 2 {\text{rl}}}} }}{3}}$$

is the effective local dipolar field [27] and $\omega_{\text{L}}$ is in the Hz-to-kHz region.

A rigorous theoretical description of the dependence of $T_{{ 1 {\text{D}}}}$ on $\tau_{\text{c}}$, which covers both weak collision ($\omega_{\text{L}} \tau_{\text{c}} < 1$) and strong collision regimes ($\omega_{\text{L}} \tau_{\text{c}} > 1$), has not yet been derived. Nevertheless, an expression which reasonably joins both regimes is [27–31]

2 $$T_{{ 1 {\text{D}}\left\langle d \right\rangle }}^{ - 1} \cong C_{{ 1 {\text{D}}}}^{2} \frac{{\tau_{\text{c}} }}{{1 + 4\omega_{\text{L}}^{2} \tau_{\text{c}}^{2} }} ,$$

where (*C*_1D_)^2^ ≈ (Δ*M*_2_)_1D_ = *M*_2rl_ − *M*_2mav_ is the difference between the second moments of the NMR spectrum in the rigid lattice *M*_2rl_ and the motionally averaged value *M*_2mav_.

Contribution to *T*_1D_^−1^, coming from the interaction of ^1^H nuclear spins and unpaired electron spins of paramagnetic defects/impurities, may be described by the expression [32–34]

3 $$T_{{ 1 {\text{D}}\langle ne\rangle }}^{ - 1} = A_{{ 1 {\text{e}}}}^{2} \frac{{\tau_{\text{ce}} }}{{1 + \omega_{\text{L}}^{2} \tau_{\text{ce}}^{2} }}$$

where *A*_1e_ is the amplitude of the fluctuating local magnetic fields induced by the electron–nuclear interaction, and *τ*_ce_ is the correlation time of this process. Therefore, the experimental dependence of the dipolar relaxation rate, *R*_1D,_ in the natrolite may be described by the expression

4 $$R_{{ 1 {\text{D}}}} = T_{{ 1 {\text{D}}}}^{ - 1} \cong C_{{ 1 {\text{D}}}}^{2} \frac{{\tau_{\text{c}} }}{{1 + 4\omega_{\text{L}}^{2} \tau_{\text{c}}^{2} }} + A_{{ 1 {\text{e}}}}^{2} \frac{{\tau_{\text{ce}} }}{{1 + \omega_{\text{L}}^{2} \tau_{\text{ce}}^{2} }}.$$

The result of the simulation of the temperature dependence of *R*_1D_ with Eq. (4) is presented in Fig. 6, which shows a satisfactory agreement between the experimental data and calculations. The obtained adjusting parameters are $C_{{ 1 {\text{D}}}} \cong 14.65$ kHz rad, *τ*_ce_ = *τ*_c0‹*d*›_ exp(*E*_a‹*d*›_/*kT*); *τ*_c0‹*d*›_ = 10^−12^ s, *E*_a‹*d*›_ = 40 kJ/mol; *A*_1e_ = 170 kHz rad, *τ*_ce_ = *τ*_c0‹*e*›_ exp(*E*_a‹*e*›_/*kT*), *τ*_c0‹*e*›_ = 10^−12^ s, and *E*_a‹*e*›_ = 840 J/mol.

To explain the observed value of *C*_1D,_ we calculated the theoretical second moment of the proton spectrum in the rigid lattice *M*_2rl_. For the calculation of the second moment *M*_2rl,_ the positional parameters for all hydrogen, Al, and Na atoms were taken from the neutron diffraction study [5]. Our calculation of the intramolecular contribution (the interaction between two protons of water molecule) to *M*_2rl_ was found to be ≈24 *G*^2^ (1 *G* = 10^−4^ T), and the intermolecular contribution (the interactions between protons of different water molecules) was equal to 0.5 *G*^2^. The contributions to *M*_2rl_ from the interaction proton—^27^Al and proton—^23^Na were found to be 0.23 and 0.22 *G*^2^, accordingly. Therefore, the full second moment *M*_2rl_ is estimated to 25 *G*^2^. Using experimental value *C*_1D,_ we obtain that (Δ*M*_2_)_1D_ = *M*_2rl_ − *M*_2mav_ ≈ 0.3 *G*^2^. Such small difference between the second moment in the rigid lattice and the second moment averaged by motion, $(\Delta M_{2} )_{{ 1 {\text{D}}}} \approx 0.3$*G*^2^, can be explained, suggesting that the molecular mobility in natrolite involves 180° flips of the water molecules. Such flipping averages only the intermolecular interactions between protons of different molecules and does not affect the intramolecular ones, making the aforementioned difference in *M*_2D_ to be very small. Therefore, our study, which involves *T*_1D_ measurements, confirms the assumption of Thompson et al. [9] that the 180° flip motion of the water molecules takes place in natrolite.

## Conclusions

By measuring the ^1^H dipolar spin–lattice relaxation time, *T*_1D,_ we have established that at *T* > 200 K, the 180° flip motion of the water molecules takes place in natrolite. The activation energy of this motion is ~40 kJ/mol. By measuring the temperature transformations of ^1^H NMR spectra, we have established that at *T* > 300 K, the diffusion of water molecules along the nano-channels is responsible for the transformation of NMR spectra into narrow doublet. The dipolar interactions with paramagnetic impurities become significant as a relaxation mechanism of the ^1^H nuclei only at temperature <250 K.
